# A seleno-hormetine protects bone marrow hematopoietic cells against ionizing radiation-induced toxicities

**DOI:** 10.1371/journal.pone.0205626

**Published:** 2019-04-29

**Authors:** Desirée Bartolini, Yanzhong Wang, Jie Zhang, Daniela Giustarini, Ranieri Rossi, Gavin Y. Wang, Pierangelo Torquato, Danyelle M. Townsend, Kenneth D. Tew, Francesco Galli

**Affiliations:** 1 Department of Pharmaceutical Sciences, University of Perugia, Perugia, Italy; 2 Department of Pathology and Laboratory Medicine, Medical University of South Carolina, Charleston, SC, United States of America; 3 Department of Cell and Molecular Pharmacology and Experimental Therapeutics, Medical University of South Carolina, Charleston, SC, United States of America; 4 Department of Biotechnology Chemistry and Pharmacy, University of Siena, Siena, Italy; Emory University, UNITED STATES

## Abstract

2,2'-diselenyldibenzoic acid (DSBA) is a chemical probe produced to explore the pharmacological properties of diphenyldiselenide-derived agents with seleno-hormetic activity undergoing preclinical development. The present study was designed to verify *in vivo* the drug’s properties and to determine mechanistically how these may mediate the protection of tissues against stress conditions, exemplified by ionizing radiation induced damage in mouse bone marrow. In murine bone marrow hematopoietic cells, the drug initiated the activation of the Nrf2 transcription factor resulting in enhanced expression of downstream stress response genes. This type of response was confirmed in human liver cells and included enhanced expression of glutathione S-transferases (GST), important in the metabolism and pharmacological function of seleno-compounds. In C57 BL/6 mice, DSBA prevented the suppression of bone marrow hematopoietic cells caused by ionizing radiation exposure. Such *in vivo* prevention effects were associated with Nrf2 pathway activation in both bone marrow cells and liver tissue. These findings demonstrated for the first time the pharmacological properties of DSBA *in vivo*, suggesting a practical application for this type of Se-hormetic molecules as a radioprotective and/or prevention agents in cancer treatments.

## 1. Introduction

Inorganic and organic forms of selenium have been investigated as pharmacological agents with applications in either cancer chemoprevention (cytoprotective effects) or therapy of drug-resistant tumors (recently reviewed in [1]). These compounds act as thiol peroxidases (TP) and/or agonists of drug metabolism genes associated with the detoxification of cellular electrophiles [2].

Recently, we demonstrated that structural modifications of the diphenyldiselenide [(PhSe)_2_] scaffold can mitigate the redox cycling activity of the Se–Se functional group, thereby lessening its cytotoxicity [3]. 2,2'-diselenyldibenzoic acid (DSBA) is the resultant molecular probe generated by this strategy, possessing *in vitro* pharmacological properties and low toxicity [3]. Its TP activity is sufficient to stimulate an adaptive stress response with increased protection against H_2_O_2_-induced injury in either murine embryonic fibroblasts or human hepatocytes. To facilitate further development of DSBA as a therapeutic adjuvant, it is important to define how the drug enacts its protective effects. In vitro findings suggested that the hormetic effects of DSBA are achieved through activation of the transcription factor NF-E2-Related Factor 2 (Nrf2) [2], step-wise influencing the expression of gene products that protect against oxidative damage. These include isoform P of the enzyme glutathione S-transferase (GSTP) [4]. GSTs are among the most abundant Cys-containing cellular proteins and were the first identified to react with Se-organic compounds, thereby promoting their metabolism [5, 6]. In this context, recent studies by some of us have demonstrated that the GSTP isoform is critical for detoxification and maintenance of redox homeostasis in cells treated with SeTP [2, 3, 7]. GSTP has been characterized as an unusual member of this family, insofar as its functions transcend detoxification and include regulation of signal transduction pathways by means of S-glutathionylation, a post-translational modification of susceptible Cys residues [8]. In this context, S-glutathionylation of estrogen receptor alpha [9], is an indication of the general importance of GST family members in controlling myeloproliferation events [10].

For these reasons and because bone marrow is a dose limiting organ for radiation exposure, we have chosen to explore the protective role of DSBA on murine bone marrow hematopoietic cells and to characterize the involvement of Nrf2 and GSTP in the pharmacology of this Se-compound. Furthermore, *in vitro* studies were extended into animals to examine whether the hormetic activity of DSBA is sufficient to prevent damage to hematopoietic stem and progenitor cells from bone marrow [11]. To place the results in context and to extend DSBA activity characterization, human liver cells were used as a comparative model. In fact, these cells can be considered “a reporter cell model” for Se-hormetic activity of DSBA and other Se-compounds [2, 3].

## 2. Materials and methods

### 2.1 Seleno-compounds

2,2'-diselenyldibenzoicacid (DSBA) was synthesized as reported in [3]. Purity >98.5%. Ebselen (E3520) and diphenyl-diselenide [(PhSe)_2_] (180629; purity 98%) were purchased from Sigma-Aldrich and all compounds were dissolved in DMSO as described in detail later (see section 2.3 and 2.4).

### 2.2 In vitro studies in human liver cell lines

HepG2 human hepatocarcinoma cells were maintained in MEM medium (Gibco, Life Technology) supplemented with 10% fetal bovine serum (Gibco, Life Technology) in the presence of 100 U/ml penicillin and 100 mg/ml streptomycin (Sigma-Aldrich, USA). HepaRG human progenitor hepatic cells (Thermo Fisher Scientific) were maintained according to the manufacturer’s recommendations. Briefly, the cells were grown in William’s E medium (Thermo Fisher Scientific) supplemented with Glutamax (Gibco), 5 μg/mL human insulin (Sigma-Aldrich) and 50 μM hydrocortisone hemisuccinate (Sigma-Aldrich) for 14 days. All cells were kept at 37°C in a humidified 5% CO_2_ cell culture incubator and were passaged using trypsin-EDTA (Euroclone).

### 2.3 Cellular thiols and glutathionylation

HepG2 and HepaRG cellular thiols were assessed by HPLC analysis with fluorescence detection after derivatization with monobromobimane (mBrB, Calbiochem). For disulfide analysis, aliquots of samples were derivatized with N-ethylmaleimide (Sigma-Aldrich) to mask reduced thiols and then dithiothreitol (DTT, Sigma-Aldrich) was used to reduce disulfide bridges, according to Rossi et al. [12]. The Cayman’s Glutathionylated protein detection kit (Cayman Chrmical, Item No.10010721) was used to assess Protein S-Glutathione (PSSG) in HepG2 and HepaRG treated with DSBA, PhSe)_2_ or Ebselen (10 μM in DMSO). The final concentration of DMSO in the cell tests was 0.001% vol/vol. The method allows a direct measurement of *S*-glutathionylated proteins in whole (permeabilized) cells by flow cytometry analysis that was performed utilizing an Attune NxT Acustic Focusing Cytometer (Thermo Fisher Scientific).

### 2.4 In vivo studies

Male C57 BL/6 mice purchased from the Jackson Laboratories (Bar Harbor, ME) and were used for *in vivo* experiments. The animals were housed five per cage in the Hollings Cancer Center AAALAC-certified animal facilities at the Medical University of South Carolina (MUSC). Animals received food and water *ad libitum*. All mice were used at approximately 8–12 weeks of age. The Institutional Animal Care and Use Committee of MUSC approved all experimental procedures used in this study.

DSBA was dissolved in DMSO and then diluted with 30% PEG2000/PBS (DMSO final concentration was less than 5%). Mice were administered with a single dose of the diluted DSBA solution at 10 mg/Kg and 50 mg/Kg via intraperitoneal injection. Control animals were treated with the vehicle. The groups of mice included 3 animals (n = 3) each and the experiments were repeated 3 times (N = 3). Mice were scarified 24 hrs after the treatment to collect blood, bone marrow (BM) and liver samples. All animal studies were approved by the Institutional Animal Care and Use Committee (IACUC) at the Medical University of South Carolina (MUSC).

### 2. 5 Whole-Body irradiation (WBI) and DSBA treatment

To investigate the protection against IR injury, the number of BM HSPCs was evaluated in animals that received a dose of 50 mg/kg DSBA 4 h before WBI exposure. Mice were exposed to 3 Gy of irradiation using a J. L. Shepherd Model 143 ^137^Cs gamma irradiator at a dose rate of 2.0 Gy/min as described previously [13]. Twenty-four hours after WBI, mice were euthanized by CO_2_ suffocation followed by cervical dislocation, and the femora and tibiae were immediately harvested from the mice for the isolation of bone marrow mononuclear cells as described below.

### 2.6 Isolation of BM mononuclear cells (BM-MNCs)

The femora and tibiae were harvested from the mice immediately after they were euthanized with CO_2_. Bone marrow cells were flushed from the bones into Hank’s buffered saline solution (HBSS) containing 2% FCS using a 21-gauge needle and syringe. Cells from at least three mice were pooled and centrifuged through Histopaque 1083 (Sigma, St. Louis, MO) to isolate bone marrow BM-MNCs as described previously [13].

### 2.7. Flow cytometric analysis of hematopoietic cells

Flow cytometry was used to analyze Hematopoietic Stem Cells (HSCs) and Progenitor cells (HPCs) as previously described [14]. Briefly, BM-MNCs were incubated with PE-conjugated antibodies against CD3e, CD45R/B220, Gr-1, Mac-1 and Ter-119 to stain the lineage-positive cells. The cells were washed with PBS and incubated with anti-CD16/CD32 antibody to block Fc receptors. Finally, the cells were stained with PE-Cy7 conjugated anti-Sca-1 and APC-H7 conjugated anti-c-kit antibodies and analyzed using a BD LSRFortessa^TM^ X-20 flow cytometer (Becton Dickinson, San Jose, CA). The data were analyzed using FlowJo software. Cells stained negative for lineage markers and c-kit but positive for Sca1 were considered as HPCs (lineage^-^/Scal1^-^/c-kit^+^cells, or LSK^−^cells) and those negative for lineage markers but positive for Sca1 and c-kit as HSC-enriched cells (lineage-/Sca1^+^/c-kit^+^ cells, or LSK cells or HSCs).

### 2.8 Flow cytometric analysis of ROS

ROS levels were measured in HSCs and HPCs by flow cytometric analysis using the fluorescent probe DCFH-DA [11]. Briefly, Lin^-^ HSPCs were loaded with 5 mM of DCF-DA and incubated at 37°C for 30 min. The levels of ROS in HSPCs were analyzed by measuring the mean fluorescence intensity of DCF-DA using a BDLSRFortessa^TM^ X-20 cell analyzer (Becton Dickinson, San Jose, CA) and FACSDiva^TM^ software. Data analysis was performed using FlowJo software (Tree Star, Ashland, OR).

### *2*.9 Colony-forming unit assay

Colony-forming unit (CFU) assays were performed by culturing the isolated BM-MNCs in MethoCult GF M3434 methylcellulose medium (Stem Cell Technologies) as described previously [15]. Colonies of colony-forming unit-granulocyte macrophage (CFU-GM) and burst-forming unit-erythroid (BFU-E) were scored on day 7, while colonies of CFU-granulocyte, -erythrocyte, -monocyte, and -megakaryocyte (CFU-GEMM) were enumerated on day 12 after incubation.

### 2.10 Immunoblot of nuclear and cytosolic proteins

Cellular extracts were prepared after 4 h of treatment with DSBA and fractionation of cytosolic and nuclear proteins that was carried out with a Thermo Scientific NE-PER Nuclear and Cytoplasmic Extraction Kit (Cat# 78833, Thermo Fisher). Protein samples were extracted using cell lysis buffer (Cell Signaling) supplemented with a cocktail of proteinase inhibitors (Sigma) and protein concentrations were determined with the Bio-Rad Dc protein assay kit (Bio-Rad Laboratories). Western blots was performed as described in [16]. Briefly, 50 μg of protein samples were resolved on 10% Mini-Protean TGX gels (Bio-Rad) and transferred onto 0.2 mM PVDF membrane (Millipore). Blots were blocked with 5% nonfat milk for 1–2 h at room temperature, then probed with primary antibodies, and incubated at 4°C overnight. Primary antibodies used were: anti-GSTP (#3369), anti-Nrf2 (#12721) and anti-aldehyde dehydrogenase-1 (ALDH1) (#12035) from Cell Signaling; heme-oxygenase 1 (HO-1) (SC-390991), anti-Nrf2 (SC-772) and Tubulin (SC-23948) from Santa Cruz Biotechnology. After extensive washing with TBST, blots were incubated with appropriate HRP-conjugated secondary antibody for 1.5 h at room temperature. Protein bands were detected using an ECL Plus Western Blot Detection System (GE Healthcare Life Science).

### 2.11 GST activity

The specific activity of the GST in bone marrow, plasma samples, HepG2 and HepaRG cells were measured as previously described in [17] using 5 mM GSH (Sigma-Aldrich, St. Louis, MO) and 0.5 mM CDNB (Merck, Darmstadt, Germany) as second substrate in 0.1M potassium phosphate buffer pH 6.5 at room temperature with the Benchmark plus microplate spectrophotometer (BioRad, Hercules, CA) by following the change in absorbance at 340 nm. The molar extinction coefficient used for CDNB conjugation was 9.6 mM^-1^cm^-1^. Enzymatic activities were calculated after correction for the non-enzymatic reaction.

### 2.12 Immunohistochemical analysis (IHC)

Hepatic Nrf2 was measured by immunohistochemistry (IHC) as previously described in [18]. Briefly, mouse liver tissues were fixed with formalin and embedded in paraffin. Tissue sections (5 μm thick) were prepared. Endogenous peroxidase activity was blocked by incubation with 3% hydrogen peroxide for 30 min and followed by heating in 1mM EDTA for antigen retrieval. The sections were then blocked with 5% normal goat serum in 0.1% Triton X-100/PBS for 1 h and incubated overnight at 4 degree with rabbit anti-human Nrf2 antibody (1:200, Santa Cruz). After wash with PBS, slides were incubated with ABC reagent (Vector) for 30 min. Immunostaining was visualized by DAB and the slides were counterstained using hematoxylin.

### 2.13 Statistics

Data (as means+/-SD) were assessed for distribution and differences between variables were assessed for statistical significance using parametric or non-parametric tests when appropriate.

## 3 Results

### 3.1 In vitro effects of DSBA on liver cell ROS and thiols

**Fig 1:**
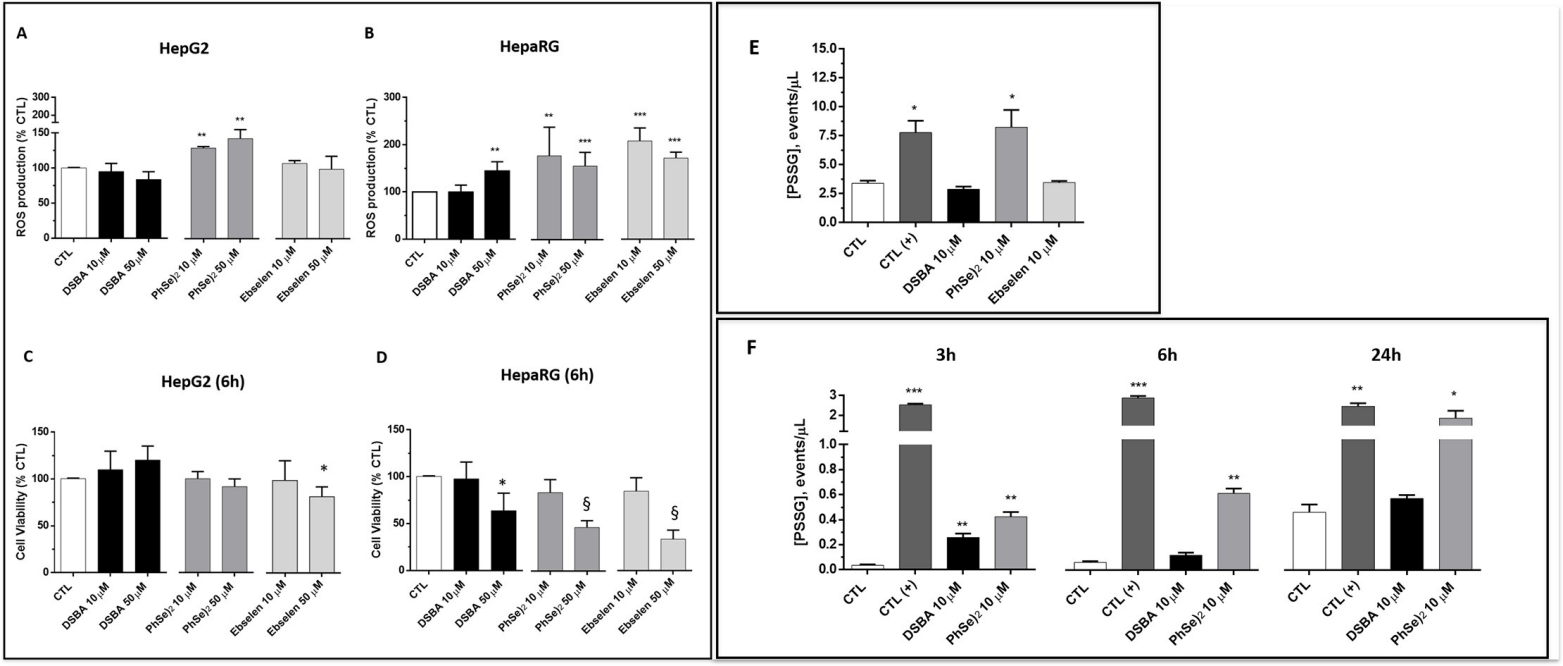
**Reactive oxygen species (A and B), cell viability (C and D) protein S-glutathionylation (E and F) in the human liver cell lines HepG2 (A, C and E) and HepaRG (B, D and F).** HepaRG or HepG2 cells were treated for 24 hours with 10 μM DSBA, PhSe)_2_ or Ebselen then ROS were measured the DCF method. Protein S-glutathionylation **(**PSSG) was assessed in permeabilized cells by FACS-Scan as described in the text.

To characterize the metabolism and effects of DSBA on cellular redox, ROS and thiol levels were assessed in two human liver cell lines, HepaRG (terminally differentiated cells) and HepG2 (hepatocellular carcinoma) after treatment with this and other Se-compounds that were used for comparison. DSBA was less efficient compared with Ebselen or the diselenide precursor (PhSe)_2_ in stimulating ROS production, which was higher in HepaRG than in HepG2 cells (Fig 1A and 1B, respectively).

In both the cell lines, treatments with DSBA and the other SeTP at 10 μM final concentration did not cause significant reductions of cell viability (Fig 1C and 1D). DSBA was not toxic at doses of up to 50 μM in these cell lines. Conversely, cell viability significantly decreased in HepaRG, but not in HepG2 cells, during the treatment with 50 μM (PhSe)_2_ or Ebselen (50% and 60%, respectively; Fig 1D). For this reason, cellular thiols and GST activity were investigated in HepaRG cells using Se-compounds only at 10 μM final concentration.

Lower fluxes of ROS in HepG2 compared with HepaRG cells might be explained by the more reduced intracellular environment of these hepatocellular carcinoma. In fact, HepG2 cells showed higher cellular levels and a lower efflux rate of GSH (Table 1), and increased GSH/GSSG ratios (256 and 170, respectively; p < 0.05). Total protein S-glutathionylation was also increased in HepG2 compared with HepaRG cells (Fig 1E vs 1F).

**Table 1:** Cellular and extracellular levels of glutathione in tumoral HepG2 and non-tumoral HepaRG human liver cell lines treated for 24 hours with DSBA.

	Intracellular GSH (nmol/10^6^ cells)	Extracellular GSH (nmol/10^6^ cells)	Intracellular GSSG (nmol/10^6^ cells)	GST activity (U/mg of protein)
**HepG2 cells**				
**CTL**	33.3 ± 5.37	2.25 ± 1.41	0.13 ± 0.03	53.40 ± 6.64
**DSBA 10 μM**	31.5 ± 5.12	3.32 ± 1.50	0.15 ± 0.05	57.20 ± 0.65
**DSBA 50 μM**	27.2 ± 6.20	2.48 ± 1.20	0.12 ±0.04	41.00 ± 10.03*
**Ebselen 10 μM**	45.0 ± 5.00	4.40 ± 1.31**	0.18 ±0.05*	23.20 ± 8.31**
**Ebselen 50 μM**	50.3 ± 4.20*	5.06 ± 2.00**	0.40 ± 0.06**	43.10 ± 2.21*
**HepaRG cells**				
**CTL**	18.70 ± 2.12	10.25 ± 3.70	0.11 ± 0.01	18.20 ± 0.63
**DSBA 10 μM**	25.60 ±4.30 *	13.85 ±5.20	0.13 ± 0.03*	6.80 ± 0.19*
**Ebselen 10 μM**	18.80 ± 5.00	16.54 ± 4.31*	0.18 ±0.07**	16.30 ± 0.07*

t-test: CTL (vehicle = DMSO) vs treatment

*p<0.05

**p<0.005

Again, in HepG2 hepatocarcinoma cells, DSBA changed neither intracellular levels of GSH/GSSG, nor the efflux of GSH (Table 1) and PSSG levels (Fig 1E and 1F). Conversely, the more potent TP compound Ebselen [2, 7] increased GSH levels and efflux in HepG2 cells, also increasing its cellular oxidation to form GSSG (Table 1). In these cells, PSSG increased only after treatment with (PhSe)_2_ (Fig 1E and 1F), i.e. the Se-compound with higher cytotoxic and TP activity in the group of molecules investigated [7].

In HepaRG cells, the Se-compounds DSBA and Ebselen both enhanced GSH metabolism, but DSBA was less efficient compared with Ebselen in stimulating GSH efflux and GSSG formation (Table 1), thereby leading to a higher GSH/GSSG ratio (from an average value of 170 in control cells to 200 in DBA treated cells and 100 in Ebselen treated cells). DSBA slightly and transiently stimulated PSSG formation in these cells that was again very sensitive to (PhSe)_2_ (Fig 1).

DSBA was less efficient than Ebselen in decreasing GST activity in the hepatocellular carcinoma cell line HepG2 (Table 1). Conversely, DSBA, but not Ebselen, reduced GST activity levels in the non-tumoral cell line HepaRG that expresses lower GST activity than HepG2 cells (Table 1). The absence of a decrease in the GST activity of HepaRG cells during treatment with Ebselen, a potent and irreversible inhibitor of GST [6], could be explained by the gene induction properties of this compound that is demonstrated for the different isoforms of GST [2]. This is also supported by the activation of other inducible processes associated with Se-compound detoxification, such as those responsible of GSH efflux (Table 1) [2]. A gene induction effect may also explain the absence of a concentration-dependent inhibition of GST activity during treatment of HepG2 cells with Ebselen.

### 3.2 DSBA activates liver tissue Nrf2 in vivo

**Fig 2:**
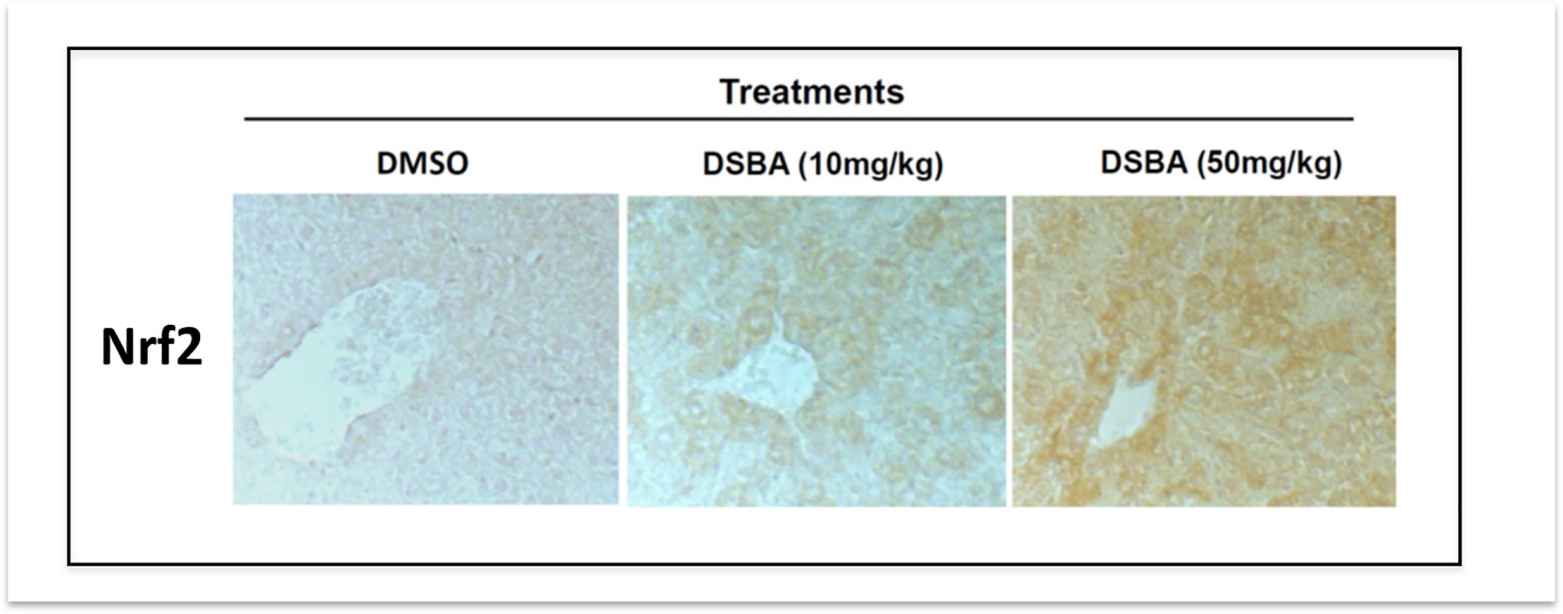
DSBA activates Nrf2 in liver tissues *in vivo*. IHC was employed to assess Nrf2 expression in liver tissues of C57 BL/6 mice at 24 h after drug treatment. Magnification 400x. Vehicle control = DMSO.

The dosages of DSBA used in this study did not cause overt toxicity as demonstrated by objective examination of animal behavior and generic symptoms, liver histology (Fig 2), blood and BM cellular composition and morphology (not shown).

At the same time, IHC analysis of mouse liver revealed that DSBA increased in a dose-dependent fashion the expression of Nrf2 protein in this tissue (Fig 2).

### 3.3 DSBA modulates the redox signaling of HSPCs in vivo

**Fig 3:**
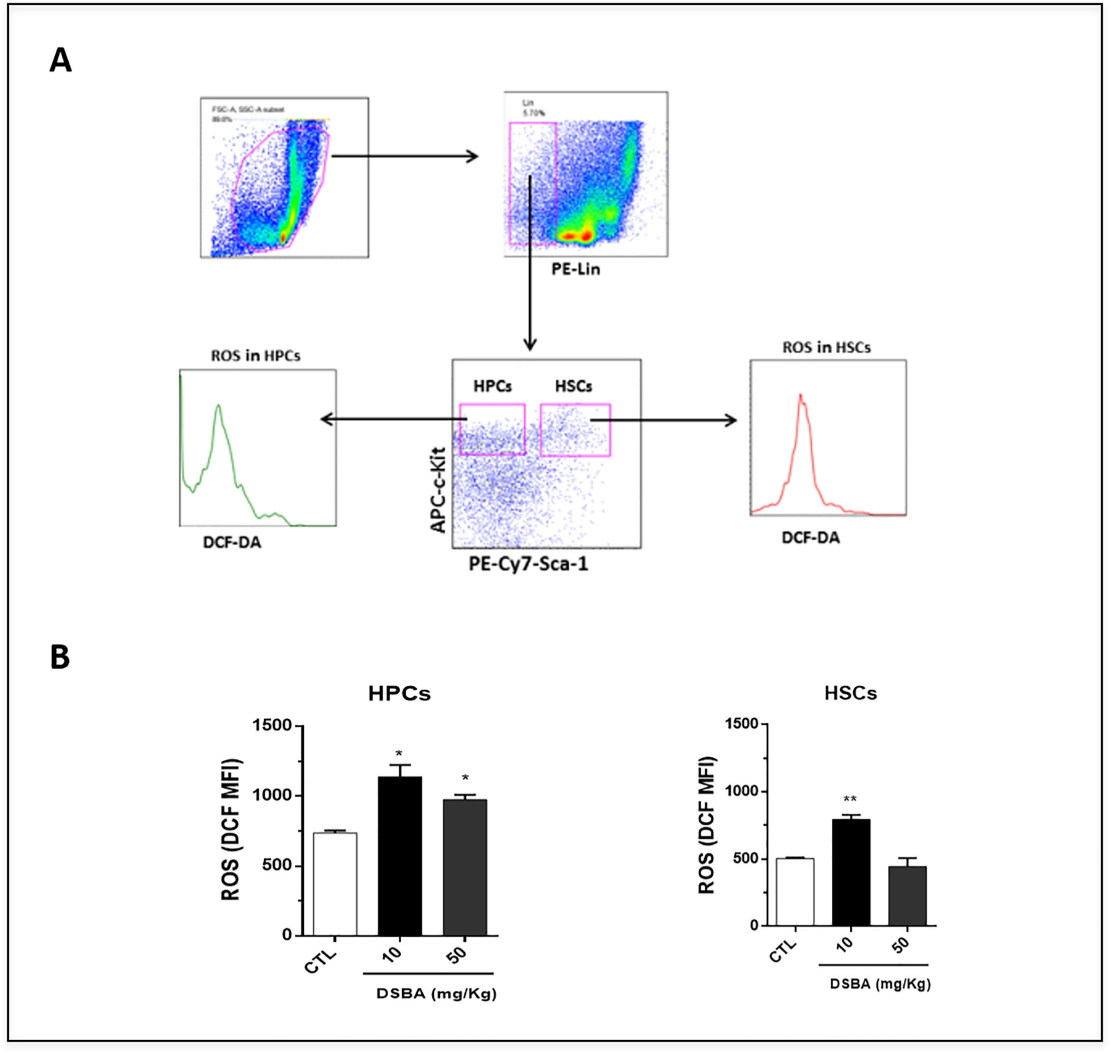
Levels of reactive oxygen species (ROS) in HPCs and HSC-enriched cells isolated from DSBA-treated C57 BL/6 mice. (**A**) BM-MNCs were collected at 24 h after DSBA treatment and subjected to immune-phenotype assays. DCF-DA staining and flow cytometric analyses were performed to measure ROS levels in LSK cells (HSC-enriched cells) and HPCs as described previously (8). Shown are representative flow cytometry graphs showing the gating strategy for measuring ROS levels in HSC-enriched cells and HPCs, respectively. (**B**) Flow cytometry assays indicated that DSBA increased ROS levels in both HSC-enriched cells and HPCs. Data are presented as mean +/- SD of three biological replicates (n = 3 mice) and analyzed by t-test: * p < 0.05, ** p < 0.01.

Previous *in vitro* studies have shown that DSBA can influence the redox of different cell types [1–3]. However, if DSBA may produce the same affect *in vivo* remains to be demonstrated. As a consequence, we investigated the impact of DSBA on ROS levels in HSPCs of C57 mice. Flow cytometry data showed that DSBA stimulates ROS generation in both HSC-enriched cells and HPCs (Fig 3); reaching peak effects at a dose of 10 mg/kg and decreasing at 50 mg/kg.

**Fig 4:**
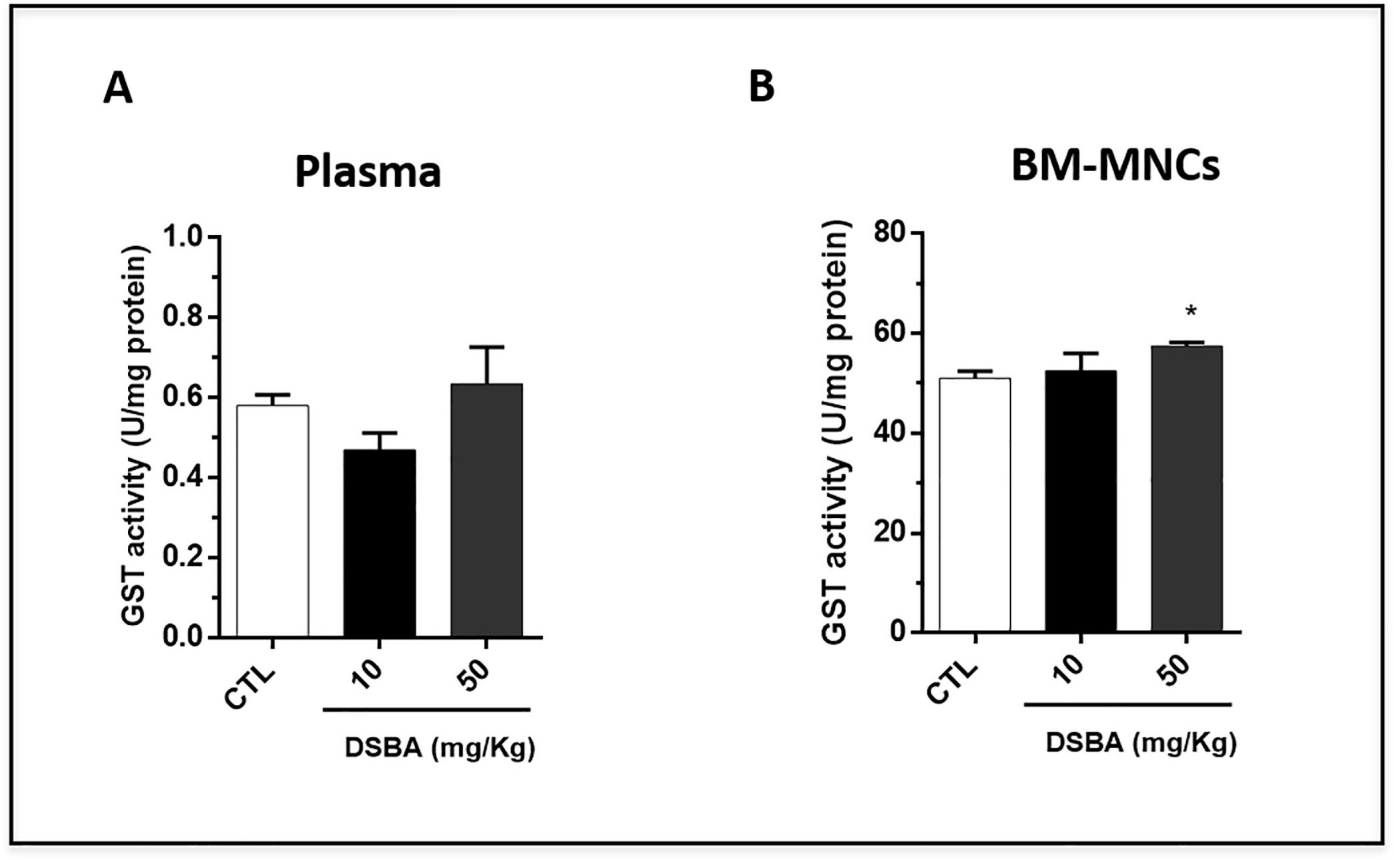
**GST activity in C57 BL/6 mouse plasma (A) and BM-MNCs (B) after DSBA treatment.** GST activity in plasma and BM-MNCs was measured at 24 h after DSBA treatment using the approaches as described in the methods section. Data are presented as mean +/- SD of three biological replicates (n = 3 mice) and analyzed by t-test: *p<0.05.

Since GST plays a significant role in restoring the redox balance of cells treated with Se-compounds [7, 19], we examined whether DSBA may affect GST activity in vivo. Our data showed that there was no significant change in plasma GST activity, a surrogate indicator of tissue GST levels, after DSBA treatment (Fig 4A). However, the enzymatic activity significantly increased in BM-MNCs obtained from animals treated with 50 mg/kg DSBA (Fig 4B). These results implied that DSBA-induced increases in ROS are not likely the consequence of GST inhibition in these cells. In contrast, DSBA-mediated ROS production may intervene in the adaptive stress response of these cells increasing GST transcription and activity, a response mechanism already described for this Se-compound in [2, 19].

### 3.4 Hematopoietic radioprotection by DSBA correlates with Nrf2 activation in BM-MNCs

**Fig 5:**
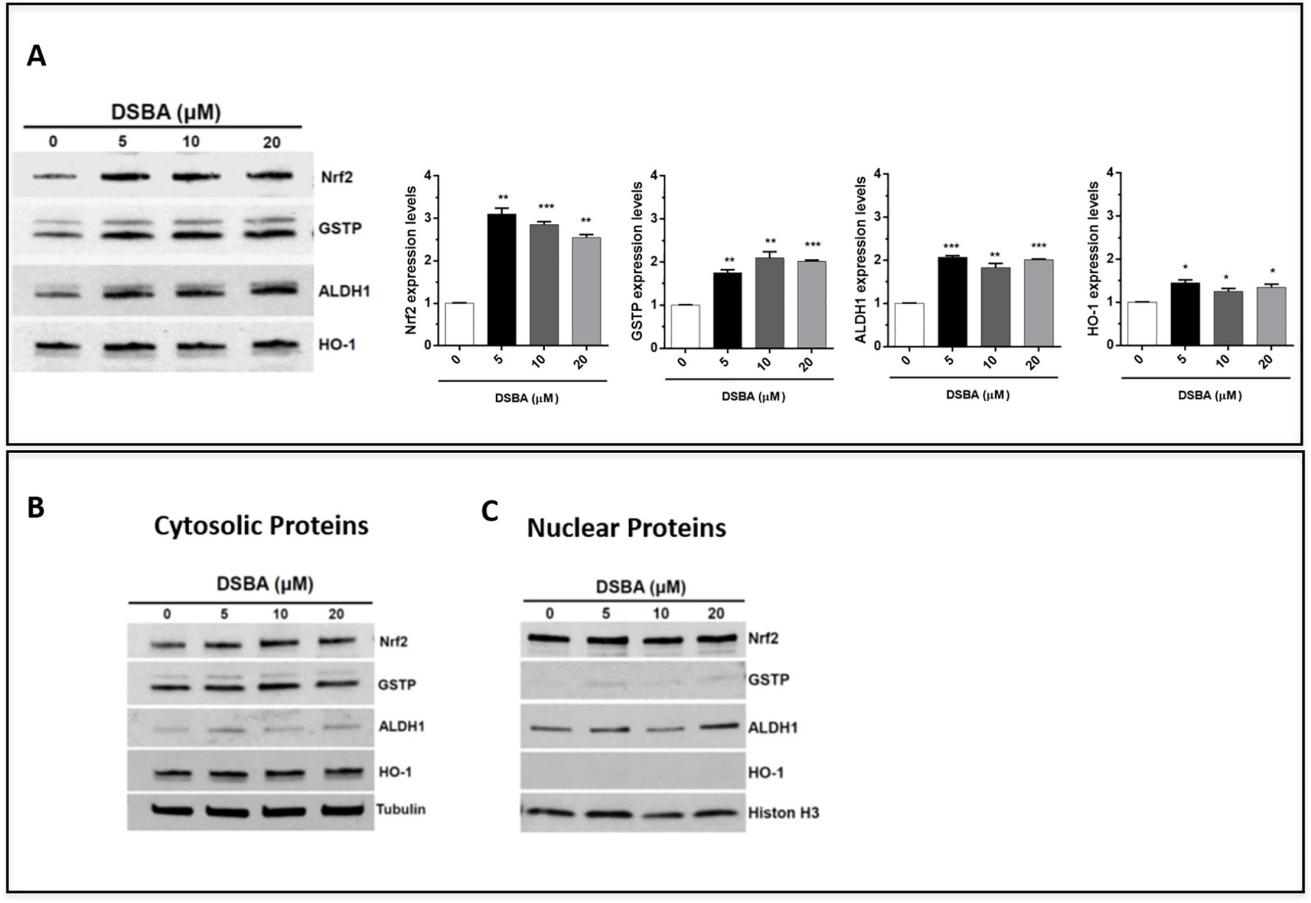
Activation and nuclear translocation of Nrf2 in DSBA-treated BM-MNCs. BM-MNCs were treated for 4h with DSBA at increasing concentrations from 5 to 20 μM and levels of Nrf2 protein and the Nrf2-dependent genes HO-1, ALDH1, and GSTP were assessed by immunoblotting in cellular extracts before (A) or after fractionation of cytosolic (B) and nuclear components (C).

To elucidate the mechanisms by which DSBA protects bone marrow hematopoietic cells against radiation injury, we determined whether this compound may impact the Nrf2-dependent signaling in these cells. Immunoblots of BM-MNC proteins further confirmed the *in vivo* effects of DSBA as an Nrf2 activator reported in [19]; besides Nrf2 protein up-regulation (compatible with the forward-feeding mechanism of regulation that characterizes this transcription factor), DSBA treatment increased the expression of GSTP as well as of other Nrf2-dependent genes, such as HO-1 and ALDH1 (Fig 5A); at the same time, Nrf2 protein levels slightly increased after DSBA treatment in the nuclear fraction of BM-MNCs (Fig 5B); ALDH1 and GSTP proteins were also present in the nucleus and their levels increased following DSBA treatment (Fig 5B).

### 3.5 DSBA protects HSPCs against radiation injury in vivo

**Fig 6:**
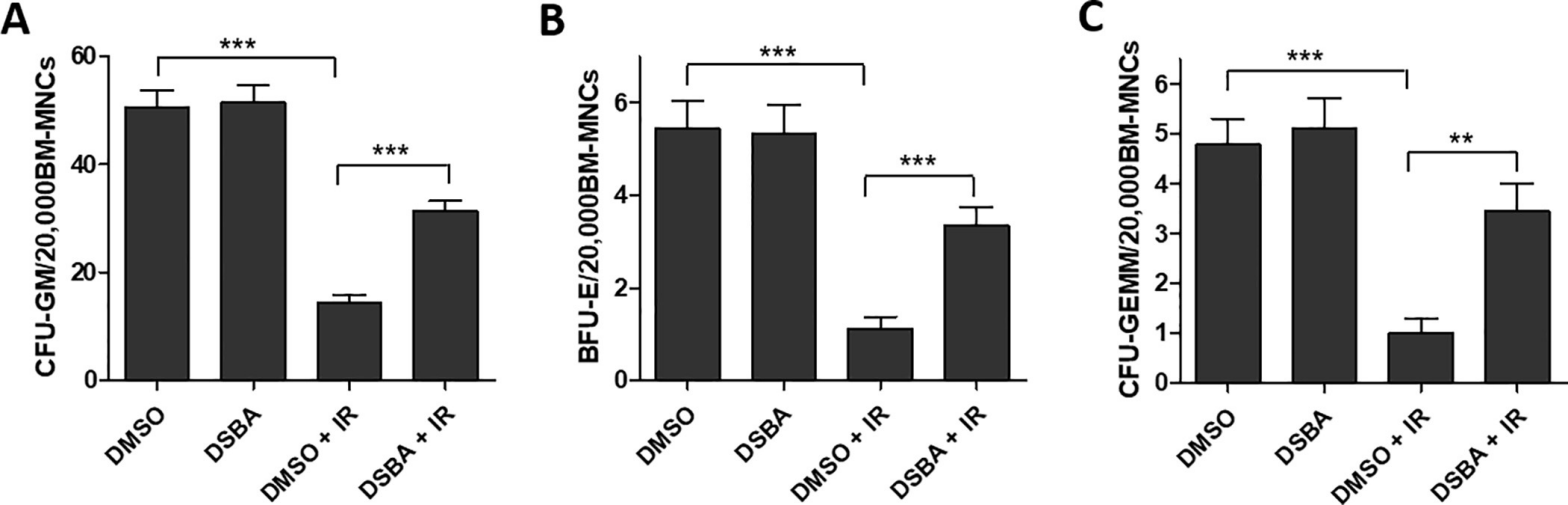
DSBA pre-treatment protects mouse BM HSPCs against ionizing radiation (IR)-induced injury in vivo. The clonogenic function of HSPCs was measured using CFU assays (12). average number of (A) CFU-GM, (B) BFU-E and (C) CFU-GEMM in 20,000 BM-MNCs. Data are presented as mean ± SEM of three independent experiments (N = 3; n = 3 mice). ** p < 0.01. *** p < 0.001.

The therapeutic potential of DSBA as a radiation protector was explored in C57 mice exposed to WBI. CFU assays were employed to measure the colony-forming capacities of HSPCs. The results showed that DSBA pre-treatment prevented the IR-induced decrease of CFU-GM, BFU-E and CFU-GEMM numbers (Fig 6), indicating that DSBA does possess radioprotective properties against IR-induced injury in HSPCs.

## 4 Discussion

We have shown in the present study that a component of the pharmacological activity of DSBA lies in the capacity of this chemical probe to activate Nrf2 and its stress response genes in bone marrow hematopoietic cells, confirmative of previous in vitro data obtained on this seleno-hormetic agent [3]. Nrf2 activation was demonstrated by assessing also liver tissue and BM hematopoietic stem cells of C57 BL/6 mice following treatment with sub-cytotoxic concentrations of DSBA. According with the canonical Nrf2 activation model, DSBA generated ROS stimulate this transcription factor (in both hepatocytes and BM) through dissolution of its interaction with Keap1, allowing migration to the nucleus and promotion of antioxidant and electrophile responsive elements [20]. Indeed, the lowest dose of DSBA investigated in this study caused such a response in BM cells, associated with nuclear translocation of Nrf2 protein and expression of a series of Nrf2-dependent genes. In these BM cells, GSTP was the most responsive gene followed by ALDH1 and HO-1; and importantly, GSTP and ALDH1 were also upregulated in the nucleus. In mice treated with 50 mg/kg DSBA, the GST gene response of BM cells was associated with ROS levels that decreased when compared with the 10 mg/kg dosage, implying that this dose was effective in stimulating an adaptive stress response.

The observation that DSBA increased hepatic Nrf2 in vivo (Fig 2) is in agreement with previous findings obtained in human liver cells [3]. Moreover, in vitro data on HepaRG cells demonstrated that the mild redox activity of DSBA is sufficient to activate Nrf2 and its downstream gene response, a key transcriptional process in the GSH metabolism of the liver cell [2, 20]. Interesting enough, these properties of DSBA did not influence the abnormal redox of HepG2 hepatocellular carcinoma cells, an effect that can be achieved with other diselenides, such as the DSBA precursor (PhSe)_2_, investigated as chemotherapy agents in drug resistant cancers [1].

At the same time, DSBA appears to be a safe Se-organic molecule since no signs of acute toxicity in the liver and BM of animals were observed after treatment, confirmative of low toxicity profile described in vitro both in this study and previous work reported in [19]. Nevertheless, further studies are needed to delineate if this agent does achieve utility as a long-term preventative drug and if any chronic adverse effects or delayed toxicities may exist.

Significantly, DSBA inhibited GST activity in HepaRG cells, concomitantly stimulating GSTP gene expression *in vivo*. This is not a trivial observation if we consider that GST is the first and likely the most important thiol-containing protein identified to react with Se-compounds; the resulting cysteine alkylation of GST protein is involved in sequestration and metabolism of Se-compounds in the liver [5, 6]. At the same time, this cysteine alkylation causes the irreversible inhibition of GST enzyme activity, a process originally described for the prototypical compound Ebselen [6] and confirmed in the in vitro experiments of this study. These effects may also help to explain the transcriptional upregulation of *GSTP*, being this an important event in drug resistance and Se-compound detoxification mechanisms [1, 21]. The expression of GSTP isoform was identified to influence the cellular response to either the para-hormetic or cytotoxic effects of redox-active Se-compounds [3][7]. Intriguingly, such a response may depend on the capability of GSTP to functionally and physically interact with Nrf2 protein (recently reviewed in [2]), a process that may depend on the capacity of this redox chaperonine to act as an S-glutathionylase enzyme [22, 23] of different cellular compartments involved in the Nrf2 transcriptional mechanism, i.e. the cytosol and the nucleus [24].

At the same time, GSTP gene expression is enmeshed in pathways that control proliferation and migration of BM myeloid cells and among these cells, the myeloid lineage is known to be highly responsive to GSTP-targeted pharmacological agents [25]. In the present report, in vivo treatment with DSBA increased GSTP expression in both the cytosol and nucleus of BM progenitor cells. This finding is in agreement with the previously reported co-localization of GSTP and Nrf2 in both the cytosolic and nuclear compartments during drug-induced activation [3]. Therefore, the nuclear availability of GSTP together with relevant concentrations of protein thiols and GSH in the nuclear environment [26], make such co-localization potentially strategic for nuclear protection and redox-dependent regulation of transcriptional processes associated with SeTP metabolism.

A main goal of this study was to determine whether DSBA may have in vivo hormetic effects. A whole body irradiation model was used for these experiments where irradiation plays a causative role in producing ROS and oxidative stress, resulting in BM stem cell damage and subsequent myelosuppression [11]. Our results conclusively demonstrated that DSBA pretreatment prevents hematopoietic stem cell damage and death in IR-exposed animals. In this regard, recent studies found that some redox-active superoxide dismutase mimetics produce similar positive effects on BM cells, behaving mechanistically in a similar fashion [27]. Therefore, there may be a possible role for DSBA in the management of radiation emergency situations or the types of hematologic toxicities observed in patients undergoing chemo-radiotherapy [14]. In this regard, Amifostine gained FDA approval as a radioprotective agent specifically preventing xerostomia during radiation treatment of head and neck tumors [28–30]. Since prevention of whole body toxicities could be an adjuvant for use of DSBA, there would be limitations inherent in the exposure timing of IR versus administration of the agent.

In conclusion, the present study described for the first time the pharmacological properties of DSBA in vivo. This molecule was demonstrated to prevent the damage of bone marrow hematopoietic cells during exposure to ionizing radiations, compatible with an efficient seleno-hormetic activity of this chemical probe prepared starting from (PhSe)_2_ scaffold and proposed before in a series of in vitro studies [2, 3]. Mechanistically, Nrf2 activation and GSTP gene induction are molecular responses associated with the pharmacological activity of DSBA in hematopoietic cells.
